# Bilayer Scaffold for Corneal Stromal Engineering: Solvent-Cast Polyvinyl Alcohol/Sodium Alginate and Electrospun Aligned Polycaprolactone Fibers

**DOI:** 10.3390/polym18151928

**Published:** 2026-08-06

**Authors:** Amin Orash Mahmoudsalehi, Kevin Stalin Catzim Rios, Carlos Enrique Guerrero-Beltrán, Wendy Ortega-Lara

**Affiliations:** 1 Department of Chemistry and Nanotechnology, School of Engineering and Science, Tecnologico de Monterrey, Monterrey 64849, Nuevo Leon, Mexico; a00818960@tec.mx; 2 School of Medicine and Health Sciences, Tecnologico de Monterrey, Monterrey 64849, Nuevo Leon, Mexico; enriqueguerrero@tec.mx

**Keywords:** corneal stromal engineering, bilayer membrane, solvent casting, electrospinning

## Abstract

Due to their limited functional range, single-layer engineered scaffolds often fall short of meeting the complex clinical requirements for corneal stromal engineering (CSE). To overcome these challenges, bilayer constructs that integrate complementary material properties have emerged as promising alternatives. In this study, we developed a bilayer membrane by electrospinning polycaprolactone (PCL) fibers onto a solvent-cast polyvinyl alcohol/sodium alginate (PVS) membrane. The dense PVS layer provided a smooth, crack-free surface with favorable physicochemical and thermal stability. In contrast, the PCL nanofibrous layer (232 ± 44 nm) exhibited a continuous, bead-free, and highly aligned morphology. Comprehensive characterization confirmed the structural integrity of the bilayer scaffold, which showed two distinct thermal transitions (~65 °C for PCL and ~225 °C for PVS), confirming good stability and minimal interfacial disruption. Functionally, the PCL–PVS bilayer scaffold demonstrated an intermediate contact angle (57.44°), high water uptake capacity (424.44%), and a high gel fraction (96.12%), along with controlled biodegradation (39.65%), highlighting its suitability for physiological environments. Mechanical testing revealed a Young’s modulus of 2.60 ± 0.20 megapascals (MPa), an ultimate tensile strength (UTS) of 5.74 ± 0.02 MPa, and an elongation at break of 3.32 ± 0.10%, values well aligned with the mechanical demands of corneal tissue. Additionally, the construct achieved 85.01% light transmittance, essential for visual clarity, and supported measurable cell viability, although additional optimization is required to further enhance cytocompatibility. These findings demonstrate that the bilayer combines structural stability, favorable physicochemical performance, transparency, and biological compatibility, positioning it as a promising platform for further optimization toward CSE.

## 1. Introduction

The cornea, located at the anterior portion of the eye, serves as a transparent and protective tissue that refracts incoming light onto the retina, enabling vision [1]. Structurally, it consists of three primary layers: the epithelium, stroma, and endothelium. Among these, the stroma constitutes nearly 85% of the total corneal thickness and is mainly composed of orthogonally arranged collagen (Col) fibrils, proteoglycans, and keratocytes [2]. The highly ordered lamellar architecture of these stromal components is crucial for maintaining both corneal transparency and mechanical stability [3]. Damage caused by trauma, infection, or degenerative diseases can disrupt this organization, resulting in stromal opacification and vision loss [4]. Corneal transplantation remains the clinical gold standard for visual restoration; however, its broad implementation is restricted by the global shortage of donor tissue, high costs, and potential immune rejection [5]. These limitations have motivated the development of alternative regenerative strategies, particularly tissue-engineered scaffolds designed to recapitulate the native stromal structure and function [6].

Among natural polymers, sodium alginate (SA) has attracted attention in corneal stromal engineering (CSE) due to its biocompatibility, hydrophilicity, and structural similarity to glycosaminoglycans, which support favorable cell–matrix interactions [7]. However, its intrinsic brittleness, rapid degradation, and limited optical clarity restrict its use as a standalone material [8]. To address these limitations, SA is often combined with other natural or synthetic polymers to improve mechanical and physicochemical properties. For instance, Tonsomboon et al. [9,10] demonstrated that embedding electrospun gelatin (Gel) nanofibers into SA–Gel hydrogels enhanced mechanical strength by nearly an order of magnitude while preserving transparency, indicating their potential as scaffolds for corneal transplantation when donor tissue is unavailable. Furthermore, Datta et al. [11] showed that SA can be formulated into crosslinked nanoparticles with tunable stability, high drug loading, and enhanced mucoadhesion, demonstrating the polymer’s versatility for ocular applications and highlighting its potential for future bioactive or drug-delivering corneal scaffolds.

Polyvinyl alcohol (PVA) is a promising candidate for CSE due to its optical transparency, flexibility, and tunable biodegradability [12]. PVA-based materials exhibit high mechanical strength, water retention, and oxygen permeability [13]. The incorporation of PVA into Col- or chitosan (CS)-based scaffolds enhances their tensile strength and light transmittance, thereby improving their suitability for corneal applications [14,15]. For example, Seyed et al. [16] showed that PVA/CS nanofibrous scaffolds support corneal epithelial cell adhesion, proliferation, and phenotype maintenance while providing mechanical stability and transparency. Similarly, Ulag et al. [17] demonstrated that 3D-printed PVA/CS corneal stroma constructs maintain optical clarity, mechanical robustness, and cell viability, highlighting the potential of PVA blends for fabricating patient-specific corneal scaffolds suitable for CSE. Recent studies have further demonstrated the potential of electrospun PVA-based and composite scaffolds for corneal tissue engineering. For example, Wu et al. [18] developed an aligned PVA/Col nanofibrous scaffold capable of mimicking the native stromal architecture and promoting cellular organization. Jung et al. [19] fabricated multilayered keratin/PVA electrospun scaffolds with enhanced physicochemical properties for corneal implantation. Likewise, Bakhshandeh et al. [20] proposed a two-part artificial cornea consisting of a PVA hydrogel integrated with a polycaprolactone (PCL) nanofibrous ring, while Mirzaeei et al. [21] developed single- and multilayered PVA/CS-based electrospun matrices for ocular drug delivery applications. These studies collectively demonstrate the versatility of PVA-based systems for ophthalmic applications while highlighting the continued need for scaffold designs that integrate structural reinforcement, optical transparency, and biomimetic architecture for CSE.

Nonetheless, while individual PVA- or SA-based systems have shown potential, few studies have systematically optimized their compositions or fabrication strategies to achieve the precise balance of transparency, mechanical stability, and hydration necessary for CSE. Moreover, most reported approaches focus on soft hydrogels or contact lenses, whereas structured, solvent-cast SA/PVA membranes remain underexplored [18,22,23,24,25]. These membranes could offer improved stability and handling properties suitable for surgical manipulation and implantation. Our previous investigations [26] and related studies have indicated that SA/PVA hybrid films fabricated via solvent casting can yield transparent, mechanically robust, and cytocompatible membranes, providing a promising foundation for CSE. However, single-layer membranes are inherently limited in their ability to replicate the hierarchical and multi-functional nature of the native stroma. The corneal stroma is a multilayered, anisotropic tissue, where each lamella is oriented to minimize light scattering while providing directional strength and guiding stromal cell alignment [27]. In contrast, oversimplified single-layer scaffolds fail to reproduce this complex structure, often resulting in inadequate mechanical integration and suboptimal cell organization.

Electrospinning has emerged as a particularly effective technique for generating aligned fibrous layers that replicate the topography of native Col fibrils [28,29,30,31]. Electrospun nanofibers made from natural and synthetic polymers—including Col, Gel, silk fibroin (SF), alginate (Alg), PVA, and PCL—have demonstrated the ability to support stromal cell adhesion, proliferation, and ECM deposition [32]. Among these, PCL stands out for its biocompatibility, FDA approval, mechanical strength, flexibility, and slow degradation rate, all of which make it ideal for fabricating the outer layer of a corneal scaffold [33]. Baker et al. [34] demonstrated that stromal cells exhibit enhanced adhesion and proliferation on PCL substrates compared to PLGA, a result attributed to the closer mechanical match between PCL and native stromal tissue. Likewise, Kruse et al. [35] reported that electrospun PCL scaffolds provided a supportive environment for cell proliferation, whereas PMMA scaffolds exhibited cytotoxicity. These findings collectively underscore the potential of aligned PCL nanofibers to guide stromal cell behavior and mimic the organized Col structure of the native stroma.

Recent studies have explored bilayer and gradient scaffolds to mimic the layered architecture of the cornea [36,37,38,39]. Esmaeili et al. [36] enhanced the mechanical strength of the amniotic membrane by coating it with Polydimethylsiloxane (PDMS), forming an ultrathin, transparent, and suturable bilayer scaffold that promoted epithelial attachment and complete corneal healing in rabbits. Nie et al. [37] developed a hydrogen–bonding–strengthened gel/carbohydrazide-modified Alg (Alg-CDH) hydrogel with sequential crosslinking, producing a tough, transparent, and suturable bilayer scaffold that supported epithelial–stromal regeneration and reduced scarring in a rabbit anterior lamellar keratoplasty (ALK) model. Wang et al. [38] fabricated a 3D-printed biomimetic limbus using gelatin methacrylate (GelMA)–poly (ethylene glycol) diacrylate (PEGDA) bioinks loaded with corneal epithelial and stromal stem cells, restoring transparency and tissue integrity in a rabbit Limbal stem cell deficiency (LSCD) model. He et al. [39] developed a 3D-printed PEGDA–GelMA bilayer corneal construct that promoted in vivo regeneration. Although these studies demonstrate advanced biofabrication and in vivo outcomes, none have combined a solvent-cast PVA/SA membrane with highly aligned electrospun PCL nanofibers while systematically evaluating the individual components and the integrated bilayer scaffold through comprehensive physicochemical, mechanical, optical, degradation, and preliminary biological characterization.

**Figure 1:**
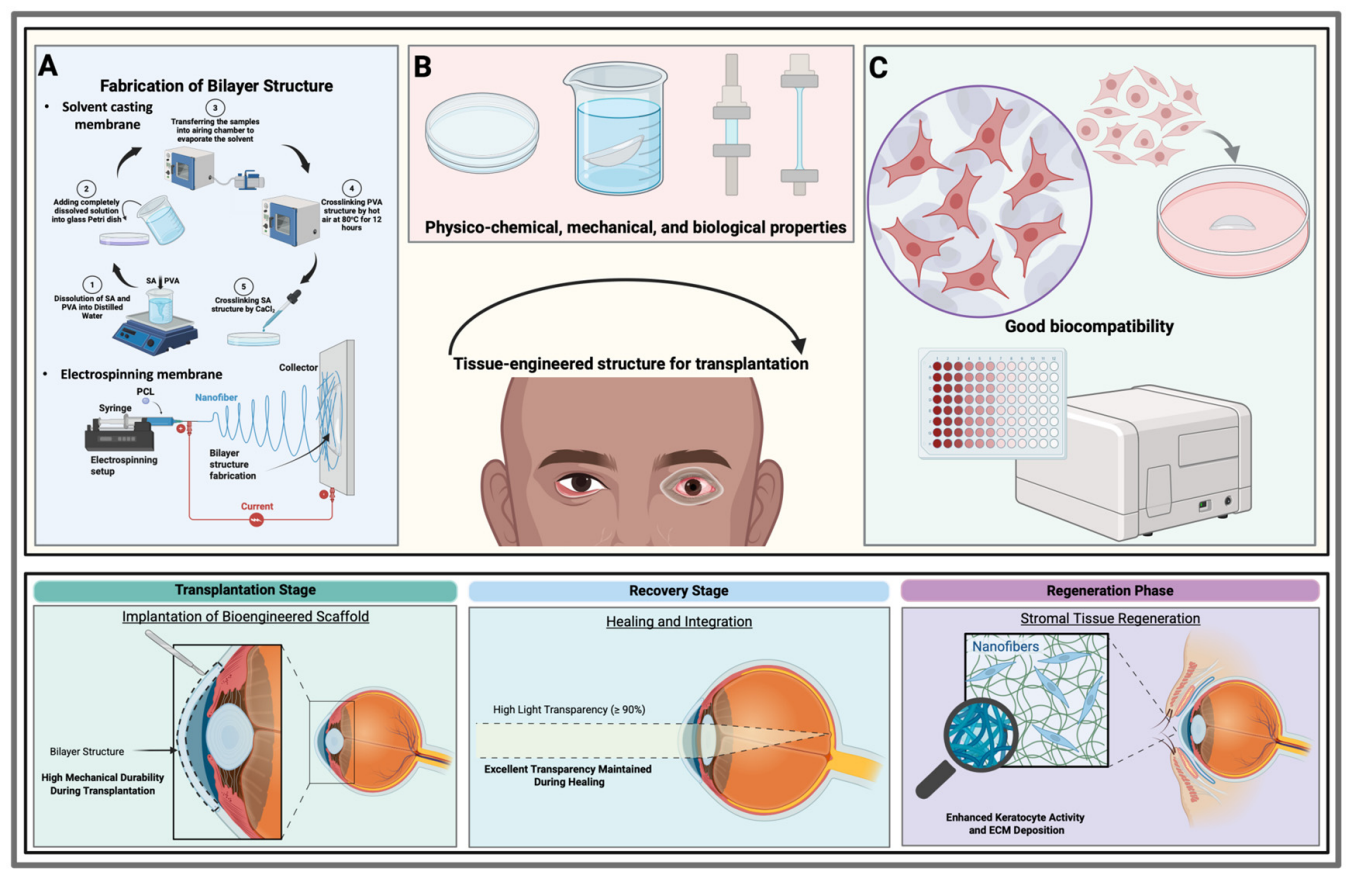
Schematic representation of the overall workflow and envisioned application of the engineered bilayer scaffold for corneal stromal engineering (CSE). (**A**) Fabrication: solvent casting forms the base layer (SA/PVA), followed by electrospinning of aligned polycaprolactone (PCL) nanofibers. (**B**) Physicochemical and mechanical characterization: structural, optical, and tensile assessments. (**C**) Biological evaluation: in vitro cell viability assessment performed in the present study. The scaffold is intended to guide the transplantation, recovery, and regeneration phases post-implantation, which represent the conceptual basis of this design and will be explored in future studies. Created in BioRender. Orash, A. (2026) https://BioRender.com/e06k851 (accessed on 3 August 2026).

Building on these principles, the present study introduces a bilayer membrane integrating the complementary advantages of both material systems: a solvent-cast SA/PVA film providing mechanical stability, optical transparency, and hydration at the base, and an aligned electrospun PCL layer supplying directional topography and structural reinforcement. A three-phase regeneration hypothesis guides this design: (1) transplantation, where mechanical integrity enables surgical handling and suture retention; (2) recovery, where transparency and hydration are maintained for optical restoration; and (3) regeneration, where aligned fibers direct stromal cell migration, elongation, and ECM remodeling toward native-like tissue integration [40]. These phases represent the theoretical foundation of the design and were not directly assessed beyond preliminary cell viability evaluation. As summarized in Figure 1, the bilayer scaffold was fabricated through a sequential process: solvent casting forms the base layer, followed by electrospinning to deposit aligned PCL nanofibers mimicking stromal lamellae. The figure also outlines physicochemical, mechanical, and biological evaluations, with in vitro cell viability as the only biological assessment in this study. This workflow embodies a biomimetic strategy bridging oversimplified single-layer constructs and the native cornea’s hierarchical architecture. By reproducing the native multilayered structure and providing guided cellular orientation, the proposed bilayer design aims to advance current CSE strategies, offering a versatile and scalable platform for developing next-generation biomimetic corneal substitutes capable of restoring both transparency and function [41].

## 2. Materials and Methods

### 2.1. Preparation and Characterization of Solvent-Cast Membranes

Solvent-cast membranes (SCMs)—PVS5, PVS7.5, and PVS10—were fabricated as the hydrophilic base layer of the bilayer scaffold. PVA and SA were dissolved in deionized (DI) water at a fixed 70:30 weight ratio, with total polymer concentrations of 5, 7.5, and 10 wt%. The mixtures were stirred at 80 °C for 4 h to ensure complete dissolution, and equal volumes were cast into flat-bottomed Petri dishes to maintain uniform thickness, a key factor influencing transparency and mechanical strength. After drying overnight, membranes underwent a two-step crosslinking process: thermal crosslinking at 80 °C overnight followed by immersion in 4 wt% CaCl_2_ solution for 5 min [26].

Morphology was observed by scanning electron microscopy (SEM; TESCAN-Vega 3, Tescan, Czech Republic) at 20 kV after sputter-coating with gold. Optical transparency was assessed on circular samples (6 mm diameter) immersed in PBS for 24 h. Transmittance (415–750 nm) was measured using a SYNERGY Mx spectrophotometer (BioTek, Winooski, VT, USA) and calculated as

T (%) = 10^−A^ × 100 (1)

where A is absorbance. Results are presented as mean ± SD (n = 3) [42].

Mechanical properties—elongation at break, Young’s modulus, and ultimate tensile strength (UTS)—were measured. Film thickness was determined by averaging three random measurements. Tensile tests were conducted using a universal testing machine (Instron, Norwood, MA, USA) with a 10 N load cell at a crosshead speed of 10 mm/min, using rectangular specimens (90 × 10 mm) [43].

The SCM, both before and after crosslinking, were characterized by Fourier transform infrared (FTIR) spectroscopy (Frontier, PerkinElmer, Norwalk, CT, USA) in the 400–4000 cm^−1^ range at room temperature to evaluate the chemical structure and to confirm the occurrence of crosslinking.

### 2.2. Fabrication and Characterization of Electrospun Fibers

Aligned electrospun membranes—PCL7.5, PCL10, and PCL12.5—were prepared as the outer structural layer of the bilayer scaffold. PCL pellets (Mn = 80,000; Sigma-Aldrich, Co., Ltd., St. Louis, MO, USA) were dissolved in chloroform (Sigma-Aldrich, Co., Ltd., St. Louis, MO, USA) at concentrations of 7.5, 10, and 12.5 wt% and stirred at 40 °C for 8 h. Electrospinning was performed using a rotating mandrel collector (7.5 cm diameter) under optimized parameters: rotation speed 1200 rpm, flow rate 0.5 mL/h, needle-to-collector distance 15 cm, applied voltage 16 kV, with a 21-gauge blunt needle and 5 mL syringe. The process was run for 1 h, yielding membranes ~20 µm thick, which were dried at 37 °C for 24 h to remove residual solvent [29].

SEM (20 kV, gold-coated samples) was used to evaluate fiber morphology. Fiber diameter and alignment were quantified from SEM images using ImageJ (version 2.0.7; Wayne Rasband, National Institute of Health, Bethesda, MD, USA) by analyzing ~100 fibers per sample (n = 3).

Transparency was measured following the same procedure deed in Section 2.1, using Equation (1) [42].

Tensile properties (elongation at break, Young’s modulus, and UTS) were determined in accordance with ASTM D638 using an Instron universal testing machine (TM-SM, Instron, High Wycombe, UK) with a 10 N load cell and a crosshead speed of 10 mm/min. Data are reported as mean ± SD (n = 3) [43].

### 2.3. Fabrication of PCL–PVS Bilayer Membrane

To mimic the native layered tissue architecture, a PCL–PVS bilayer scaffold was assembled by combining solvent-cast and electrospun layers. The inner hydrophilic layer (PVS7.5) was prepared as described in Section 2.1, while the outer fibrous layer (PCL10) was deposited directly onto the crosslinked PVS7.5 film by electrospinning under the conditions described in Section 2.2. The Bilayer constructs were collected on aluminum sheets and vacuum-dried at 37 °C for 24 h to remove residual solvents.

### 2.4. Characterization of PCL–PVS Bilayer Membrane

#### 2.4.1. Morphological Assessment

Bilayer morphology and microstructure were observed by SEM (10 kV, gold-coated samples). Fiber diameter and alignment were measured from images using ImageJ (version 2.0.7; Wayne Rasband, National Institutes of Health, Bethesda, MD, USA).

#### 2.4.2. Chemical Analyses

FTIR spectroscopy was performed on PVS7.5, PCL10, and the bilayer in the 400–4000 cm^−1^ range at room temperature. DSC (DSC200F3, Netzsch-Gerätebau GmbH, Selb, Germany) was used to assess thermal behavior. Samples (5–10 mg) were sealed in aluminum pans and heated from room temperature to 350 °C at 10 °C/min under nitrogen (10 mL/min) [44].

#### 2.4.3. Surface Wettability

A contact angle meter measured Static water contact angles (WCA) (OCA 15EC, DataPhysics, Stuttgart, Germany). A 4 µL DI water droplet was placed on the sample surface, and the angle was recorded after 30 s. Results represent mean ± SD (n = 3).

#### 2.4.4. Water Uptake Capacity

Immersing samples in DI water evaluated swelling behavior at 37 °C for up to 72 h. Samples were removed, blotted, and weighed at 3, 24, 48, and 72 h. Water uptake (U%) was calculated as

U (%) = (W_t_ − W_d_)/W_d_ × 100 (2)

where W_t_ is the wet weight and W_d_ is the initial dry weight. Thickness was also recorded to monitor dimensional changes.

#### 2.4.5. Gel Fraction

Crosslinking efficiency was determined by gel fraction. Samples were dried at 60 °C to constant weight (W_d1_), immersed in DI water for 24 h, rinsed, and redried to obtain W_d2_. The gel fraction was calculated as

Gel Fraction (%) = W_d2_/W_d1_ × 100 (3)

Values represent mean ± SD (n = 3) [45].

#### 2.4.6. Biodegradation Study

Samples were immersed in DI water at 37 °C for 28 days. At days 1, 3, 7, 14, 21, and 28, samples were removed, oven-dried at 40 °C for 24 h, and weighed. Degradation (ΔW%) was calculated as

ΔW (%) = (W_0_ − W_d_)/W_0_ × 100 (4)

where W_0_ is the initial dry weight and W_d_ is the post-immersion weight. Results are expressed as mean ± SD (n = 3).

#### 2.4.7. Mechanical Properties

Tensile strength, elongation at break, and Young’s modulus were measured using an Instron universal testing machine (TM-SM, Instron, High Wycombe, UK; 10 N load cell, crosshead speed 10 mm/min). Rectangular strips (90 × 10 mm) were tested under ambient conditions [43].

#### 2.4.8. Optical Properties

Transparency of PVS7.5, PCL10, and the bilayer was determined as in Section 2.1, using Equation (1). Measurements were made on circular specimens (6 mm) immersed in PBS for 24 h [42].

#### 2.4.9. Cytotoxicity Assays

Biocompatibility was tested with SVEC 4-10 endothelial cells (ATCC, Rockville, MD, USA). The samples were UV-sterilized for 30 min per side and preconditioned in medium for 24 h. After 72 h co-incubation in 96-well plates, cell viability was measured by Alamar Blue assay, recording fluorescence at 560 nm (excitation) and 590 nm (emission) using a BioTek spectrophotometer (Winooski, VT, USA) [26,46].

#### 2.4.10. Statistical Analysis

All experiments were conducted in triplicate unless otherwise noted. Data are expressed as mean ± SD. One-way ANOVA was performed using IBM SPSS Statistics version 29.0 (IBM Corp., Armonk, NY, USA), with significance at *p* < 0.05.

## 3. Results

### 3.1. Solvent Casting Membrane

Both schematic and spectroscopic analyses were employed to understand the structure–property relationship of the solvent-cast PVS membranes. As it is described, the fabrication process involved homogeneous mixing of SA and PVA, followed by casting, thermal drying, and ionic crosslinking to form stable membranes. This multi-step process was designed to promote uniform composition and controlled thickness, both of which are critical for achieving desirable optical and mechanical performance [47].

**Figure 2:**
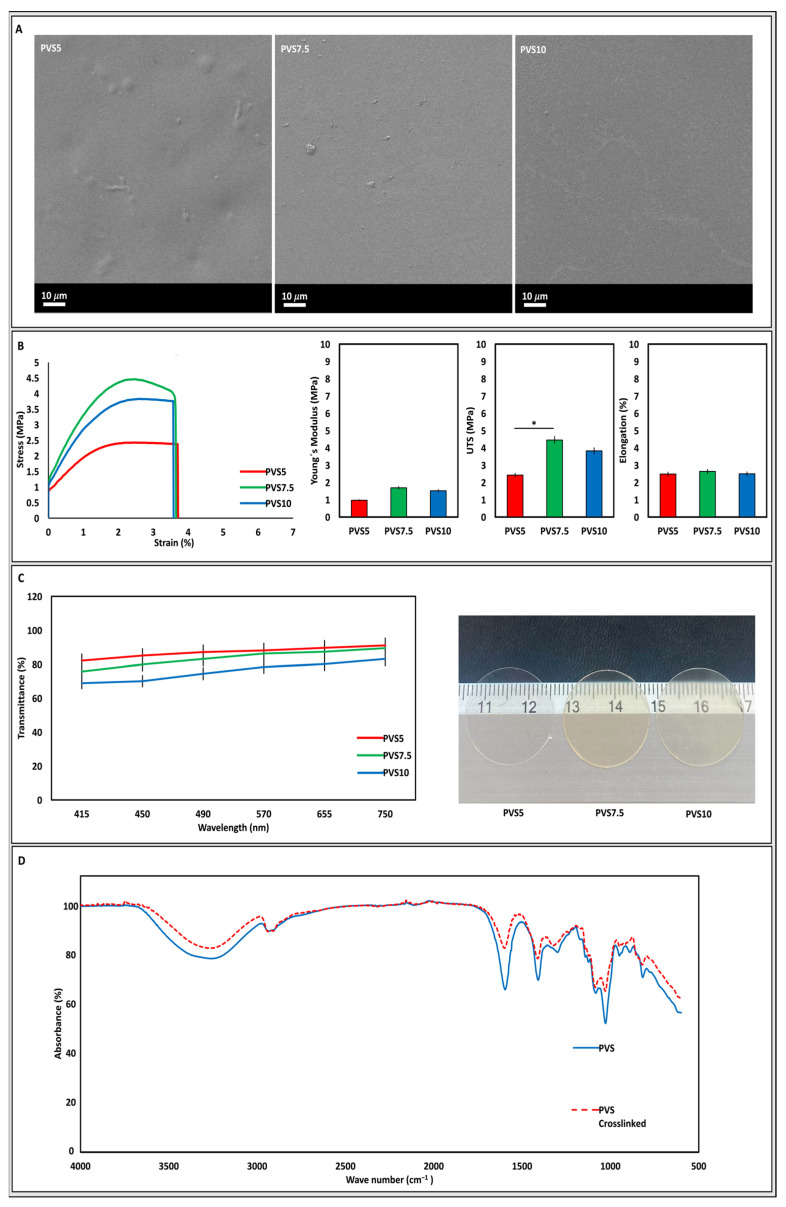
Comprehensive characterization of the polyvinyl alcohol/sodium alginate (PVS) blended membranes, highlighting their fabrication, structural features, molecular interactions, and functional properties. (**A**) scanning electron microscopy (SEM) images of the solvent-cast membranes (SCMs) show smooth and homogeneous surfaces without visible pores or defects. (**B**) Mechanical properties of PVS5, PVS7.5, and PVS10 films. The observed variations in Young’s modulus, ultimate tensile strength (UTS), and elongation are primarily attributed to differences in membrane thickness, which increased with higher polymer content (n = 3). (**C**) Optical transmittance spectra of the membranes in the 415–750 nm range, where reduced transparency at higher polymer loadings is mainly related to increased thickness and light scattering (n = 3). (**D**) Fourier transform infrared (FTIR) spectra of the PVS blended membranes before and after crosslinking. The non-crosslinked membrane (solid blue line) shows characteristic hydroxyl (–OH) and carboxyl (–COOH) peaks, while the crosslinked membrane (dashed red line) exhibits shifts and intensity changes corresponding to hydrogen bonding and calcium ion-mediated ionic interactions between PVA and SA chains. * *p*  <  0.01.

The surface morphology observed in Figure 2A indicates that the membranes maintained continuous, non-porous structures without visible defects, suggesting successful blending and film formation. The results are consistent with those reported by Dong et al. [48], who presented SEM images of the PVA–SA blended composite membrane. These images clearly show that the membrane surface is smooth, free of pores or cracks, while the internal structure is homogeneous without visible pores or signs of microphase separation. This confirms strong intermolecular compatibility between PVA and SA, leading to uniform PVS blended films with good interfacial miscibility.

The mechanical properties of SCM with different polymer concentrations—PVS5, PVS7.5, and PVS10—were evaluated under dry conditions, as shown in Figure 2B and Table 1. Although the PVA/SA weight ratio remained constant at 70/30, increasing the total polymer content led to thicker membranes (PVS5: 92 ± 6 µm; PVS7.5: 106 ± 7 µm; PVS10: 121 ± 7 µm). This thickness variation was the main factor influencing both mechanical performance and optical properties, as thicker membranes exhibited denser network structures, modified stress distribution, and greater light scattering [49,50].

Among the three compositions, PVS7.5 exhibited the highest Young’s modulus (1.81 ± 0.02 megapascals (MPa)), UTS (4.45 ± 0.05 MPa), and elongation (2.48 ± 0.4%), indicating an optimal balance between tensile strength, flexibility, and thickness. The thinner PVS5 film displayed lower mechanical performance. In contrast, the thickest PVS10 film, despite increased stiffness, showed reduced extensibility and slightly lower uniformity in mechanical response, likely due to internal structural heterogeneity arising from slower solvent evaporation and incomplete polymer chain relaxation [51]. Comparable effects of thickness on mechanical performance were reported by Zhang et al. [52], who observed that increasing the thickness of microfibrillated cellulose (MFC) films significantly enhanced tensile stress and elongation, confirming the strong influence of film thickness on polymer mechanics.

This can be attributed to less efficient solvent evaporation in thicker films, which often causes uneven drying, localized plasticization, or microvoid formation—phenomena that compromise polymer chain entanglement and lead to reduced tensile integrity [53]. Experimental studies on solvent-cast and cast-dry polymer films have demonstrated that drying dynamics, film formation kinetics, and thickness-dependent solvent retention govern the development of these stresses and defects, ultimately reducing tensile strength and elongation when thickness exceeds an optimal range [54]. In support of this, Jansson et al. [53] observed that the tangent E-modulus of starch-based films initially increased with film thickness but decreased for films thicker, highlighting that molecular orientation, relaxation, and crystallization during drying strongly influence mechanical properties. Thin films dried quickly, inducing molecular stretching and orientation, whereas thicker films allowed sufficient time for polymer chains to relax, reducing stiffness. This study emphasizes the critical role of drying kinetics and film thickness in defining the mechanical behavior of biopolymer films. Similar observations have been reported in solvent-cast biopolymer films, where increasing thickness beyond an optimal threshold reduced tensile strength and elongation due to slower evaporation kinetics and residual stress accumulation [50,51].

Interestingly, a moderate increase in film thickness may also decrease the solvent loss rate during drying, promoting molecular relaxation and reducing surface stresses; however, beyond a critical thickness, this benefit is outweighed by the formation of internal stress gradients and reduced structural homogeneity, ultimately lowering mechanical performance [51].

These findings are consistent with reports showing that moderate incorporation of SA into PVA matrices improves the mechanical behavior of hybrid casting films, while higher SA ratios may induce microphase separation or hinder effective crosslinking [48]. This trend reinforces the importance of compositional optimization for achieving scaffolds with suitable mechanical robustness for CSE applications.

Future investigations could benefit from employing innovative crosslinking chemistries and fabrication methodologies aimed at improving mechanical resilience while preserving high optical transparency. In a related study, Wu et al. [18] showed that integrating PVA into Col-based matrices enhanced mechanical performance, with a formulation containing 9% PVA–Col reaching strength values comparable to native corneal tissue. Reported mechanical characteristics of corneal tissue vary widely due to anisotropy, experimental conditions, and donor variability, but the values in Wu’s study were regarded as optimal for mimicking native stromal biomechanics [55]. Wu et al. [18] also emphasized that polymer composition, microstructure, and interfacial bonding are key parameters governing scaffold strength.

Our results follow a similar pattern: adjusting total polymer concentration (and thus membrane thickness) in PVA-based SCMs significantly modulated mechanical behavior. Therefore, film thickness acts as a double-edged factor—moderate increases enhance cohesion, while excessive thickness reduces solvent diffusion and chain mobility, decreasing tensile uniformity and overall mechanical performance [49,50].

An optimal SA level further enhanced tensile strength and flexibility, whereas excessive SA loading reduced both parameters. These outcomes agree with previous conclusions that a balanced polymer ratio is essential to achieve mechanical durability and biomedical compatibility [56].

In this study, the optical transmittance of the SCMs was assessed over the visible wavelength range (415–750 nm; Figure 2C). The observed differences in transparency correlate with membrane thickness: PVS5 (thinnest) exhibited the highest transparency (91.05%), followed by PVS7.5 (89.45%), and PVS10 (83.14%). These results are consistent with previous findings that increasing film thickness typically reduces light transmittance due to enhanced light scattering and internal reflection, leading to decreased optical uniformity [50,51]. In our samples, this trend resulted in a clear inverse correlation between thickness and transmittance, while the PVS7.5 membrane provided an optimal compromise between mechanical strength and optical transparency for corneal applications [26].

In contrast, the PVS10 film demonstrated a pronounced reduction in clarity, suggesting that excessive SA content and thickness may impair visual quality. Wu et al. [18] similarly reported that higher polymer concentrations in PVA-Col scaffolds decreased transparency due to microstructural densification, while moderate polymer loading (~7%) yielded superior optical performance. Likewise, Farasatkia et al. [57] observed that transparency in silk–GelMA casting films strongly depended on polymer formulation. Together, these results highlight the importance of fine-tuning polymer ratios to balance transparency and mechanical robustness in CSE.

FTIR analysis further elucidated the chemical interactions, reinforcing the membrane’s structure (Figure 2D). These involve hydrogen bonding between PVA –OH and SA –COOH groups, along with ionic coordination between Ca^2+^ ions and alginate carboxylates. The combined effect of thermal and ionic crosslinking yielded both intra- and intermolecular stabilization within the matrix.

FTIR spectra (400–4000 cm^−1^) confirmed crosslinking-induced structural changes: the O–H stretching band exhibited a clear shift, reflecting reduced free –OH groups and stronger hydrogen bonding, while new COO^−^ stretching peaks around 1647 cm^−1^ appeared, indicative of calcium-mediated ionic interactions. These findings agree with Chhatri et al. [58], who reported the formation of a stable polymeric network via Ca^2+^–Alg coordination. The spectra also revealed subtle changes in C–O–C and C=O bands, confirming new bond formations and crosslinking between the PVA and SA chains [59]. Collectively, these chemical modifications confirm the establishment of a robust crosslinked network, which enhances mechanical strength and aqueous stability—features essential for CSE applications.

### 3.2. Electrospun Fibers

**Figure 3:**
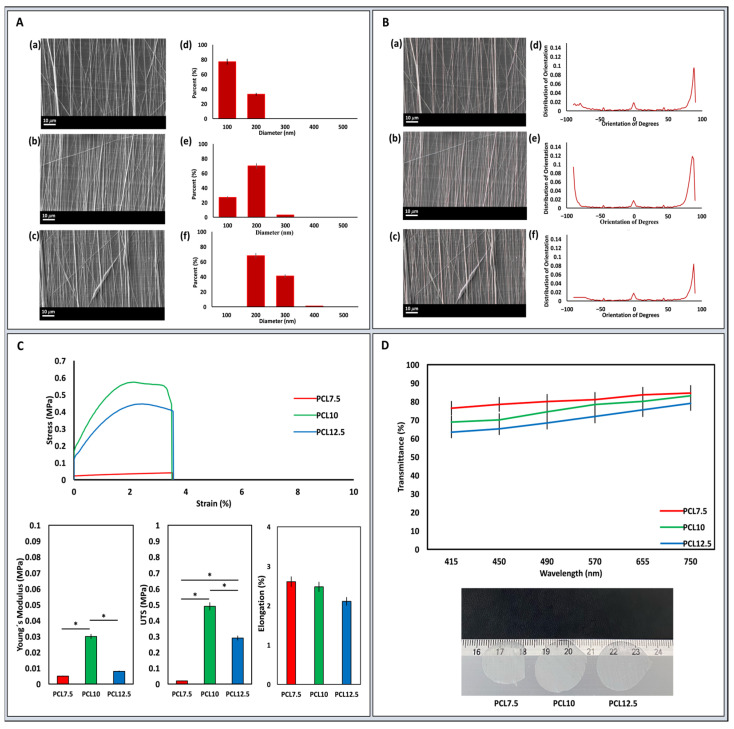
Structural, optical, and mechanical characterization of electrospun PCL scaffolds prepared at different polymer concentrations. (**A**) Morphological evaluation of electrospun fibers: (**a**–**c**) SEM micrographs and (**d**–**f**) corresponding fiber diameter distributions for (**a**,**d**) PCL7.5, (**b**,**e**) PCL10, and (**c**,**f**) PCL12.5. (**B**) Fiber alignment analysis: (**a**–**c**) color-coded orientation maps generated using OrientationJ and (**d**–**f**) angular distribution histograms for (**a**,**d**) PCL7.5, (**b**,**e**) PCL10, and (**c**,**f**) PCL12.5. (**C**) The stress–strain curves of PCL scaffolds highlight variations in mechanical behavior with different polymer concentrations (n = 3). (**D**) Optical transmittance of the electrospun scaffolds was measured across visible wavelengths (415–750 nm), indicating the influence of polymer concentration on transparency (n = 3). * *p*  <  0.01.

The second layer of the bilayer scaffold was fabricated using electrospinning, where nanofibrous structures were produced from PCL solutions at concentrations of 7.5%, 10%, and 12.5% (*w*/*v*) for evaluation before incorporation into the bilayer structure. As shown in Figure 3A, Mean fiber diameters for the PCL7.5, PCL10, and PCL12.5 scaffolds were 185 ± 43 nm, 215 ± 48 nm, and 291 ± 42 nm, respectively. The increase in fiber diameter with higher polymer concentration is consistent with established electrospinning behavior, where elevated viscosity reduces elongational deformation of the polymer jet under a constant electric field, resulting in thicker fibers [60]. It should be noted that PCL12.5 exhibited larger fiber diameters than the other samples, and its morphology lacked the uniformity and alignment observed in PCL10, likely due to reduced jet stretching and instability of the Taylor cone at higher solution viscosities [61]. As shown in Figure 3B, the morphological characterization revealed that the PCL10 formulation produced the most uniform and aligned fibers. Ghoberia et al. [62] reported that increasing the PCL polymer concentration led to a statistically significant and progressive increase in fiber diameter, ranging from 131 nm to 1173 nm. Quantitative fiber orientation analysis, performed using the OrientationJ plugin in ImageJ, color-coded directionality to visualize alignment—the same color indicated fibers sharing similar orientations. PCL10 exhibited the highest fiber alignment among the evaluated formulations, suggesting an optimal balance between electrospinning parameters and solution properties. Specifically, this concentration appeared to promote stable jet formation and consistent fiber deposition, facilitated by favorable polymer–solvent interactions and sufficient chain entanglement [28,29,30,31]. Ghoberia et al. [62] demonstrated that increasing polymer concentration can enhance fiber alignment. However, achieving the desired alignment at lower concentrations can provide comparable or even better results than higher concentrations, while avoiding the drawbacks associated with excessive polymer loading. It is important to note that higher concentrations, although potentially improving alignment, also increase fiber diameter, which may negatively affect scaffold properties. In our study, the highest PCL concentration resulted in larger fibers with reduced alignment compared to the optimal PCL10, highlighting the need to balance polymer concentration to achieve both suitable fiber diameter and alignment. All measurements are reported as mean ± standard deviation (n = 3), with statistical significance assessed via one-way ANOVA (*p* < 0.05).

Mechanical performance, a critical parameter for load-bearing and structurally stable scaffolds in corneal applications, was assessed via stress–strain analysis (Table 2; Figure 3C). PCL10 exhibited the best mechanical performance, with a UTS = 0.49 ± 0.01 MPa, Young’s modulus = 0.03 ± 0.001 MPa, and elongation = 2.58 ± 0.2%, indicating a favorable balance between stiffness and extensibility. This can be attributed to its uniform fiber network and enhanced intermolecular cohesion. In contrast, PCL7.5 showed lower stiffness (Young’s modulus = 0.005 ± 0.001 MPa) and tensile strength (UTS = 0.02 ± 0.001 MPa), but the highest elongation (2.97 ± 0.1%), characteristic of a soft, ductile structure with limited load-bearing capacity. PCL12.5 displayed intermediate mechanical properties (Young’s modulus = 0.008 ± 0.001 MPa; UTS = 0.29 ± 0.01 MPa; elongation = 2.11 ± 0.1%), possibly due to poor fiber–fiber adhesion and heterogeneous morphology arising from excessive viscosity [31]. Thus, increasing PCL concentration from 7.5% to 10% improved mechanical performance, but further increases reduced it. Ghoberia et al. [62] reported that increasing fiber diameter results in a progressive and significant decrease in the tensile strength of fibrous meshes, from 58 MPa to 31 MPa. In addition, fibers with higher alignment exhibited superior mechanical properties compared to random fibers; therefore, the higher degree of fiber alignment observed in PCL10 could also contribute to its improved mechanical performance. These findings are consistent with those reported by Wong et al. [63] and Kim et al. [64]. Furthermore, comparison with previously reported electrospun PCL-based scaffolds indicates that the mechanical properties obtained for PCL10 are consistent with the ranges reported for electrospun scaffolds intended for CSE [18,63,64]. Variations in tensile strength and Young’s modulus among different studies are expected because they depend on polymer concentration, fiber diameter, fiber alignment, electrospinning parameters, and mechanical testing conditions. In particular, aligned nanofibrous scaffolds generally exhibit superior tensile properties compared with randomly oriented fibers due to more efficient stress transfer along the fiber axis [63,64]. Therefore, the mechanical performance achieved by the PCL10 scaffold, together with its uniform fiber morphology and high degree of alignment, supports its suitability as the reinforcing layer of the proposed bilayer scaffold.

Optical transmittance was measured across the visible spectrum (415–750 nm), and the results are shown in Figure 3D. Despite PCL’s intrinsic opacity, modulation of polymer concentration and fiber architecture yielded measurable improvements in transparency. PCL7.5 demonstrated the highest light transmittance (84.61%), followed closely by PCL10 (83.14%) and PCL12.5 (79.03%). The superior transparency of PCL7.5 can be attributed to its thinner fiber diameter and lower density, which collectively reduce scattering. Himmler et al. [60] reported that light transmission through nanofibrous PCL scaffolds decreases with increasing thickness, with transmittance dropping to around 75% for scaffolds of approximately 10 µm, indicating that thicker mats intensify light scattering and reduce clarity. Similarly, Stafiej et al. [65] demonstrated that hydrogel composites reinforced with PCL nanofibers maintained high transparency, with transmittance around 80% at 750 nm. Adjusting the fiber diameter and the surrounding medium can decouple the effects of material and structural properties on optical performance [33]. For improved transparency in nanofibrous scaffolds, it is recommended to employ thin fibers and ensure that the refractive index of the scaffold matches that of its environment [28,29,30,31]. However, the mechanical limitations of this formulation constrain its practical application. Although none of the formulations matched the ~90% light transmission of the native cornea at 750 nm, PCL10 presented a favorable compromise between transparency and structural performance. The relatively high clarity of PCL10, despite its intermediate fiber diameter, highlights the importance of polymer concentration in tuning optical behavior while maintaining scaffold integrity.

Collectively, these findings identify PCL10 as the most promising formulation for the electrospun layer of the bilayer scaffold. Its superior fiber alignment, balanced optical properties, and enhanced mechanical strength suggest that it can effectively mimic the biomechanical and optical behavior of native stromal tissue—essential for maintaining corneal curvature, transmitting light, and supporting cellular attachment and proliferation in CSE. Future investigations may explore functionalization strategies or the incorporation of bioactive cues further to improve cellular responses and integration within corneal models.

### 3.3. PCL–PVS Bilayer Membrane

#### 3.3.1. Structural, Chemical, and Thermal Analysis of the Bilayer Scaffold

**Figure 4:**
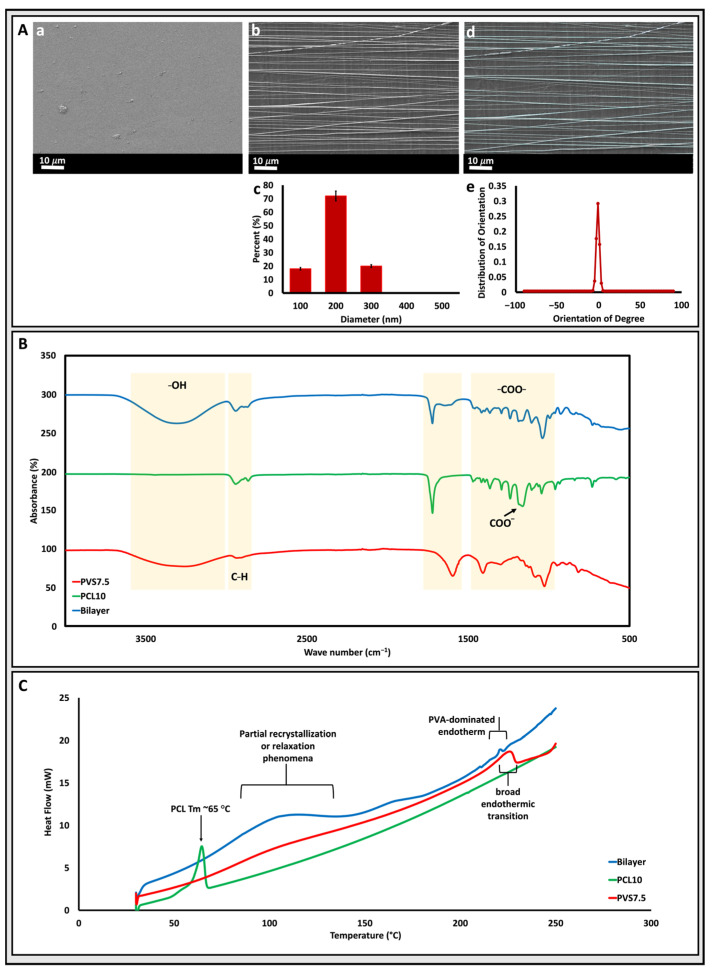
Morphological, Chemical, and Thermal Characterization of the Bilayer Scaffold. (**A**) SEM micrographs showing (**a**) the smooth and homogeneous surface morphology of the solvent-cast PVS7.5 membrane, (**b**) the continuous, bead-free, and well-aligned electrospun PCL10 nanofibers, (**c**) corresponding fiber diameter distributions, (**d**) Fiber alignment analysis; color-coded orientation maps generated using OrientationJ, and (**e**) angular distribution histograms of PCL10 fibers. (**B**) FTIR spectra of the individual layers—PVS7.5 membrane (red), electrospun PCL10 fibers (green), and the Bilayer scaffold (blue). Key absorption bands characteristic of each polymer are retained in the Bilayer, indicating physical coexistence without chemical bonding or structural alteration. (**C**) DSC thermograms depicting thermal transitions of PVS7.5 (red), PCL10 (green), and the Bilayer scaffold (blue). The bilayer displays distinct melting peaks corresponding to both components, confirming their thermal stability and compatibility within the composite scaffold.

The bilayer scaffold, composed of electrospun PCL fibers deposited onto a solvent-cast PVS membrane, was comprehensively characterized to assess its morphology, chemical composition, and thermal properties (Figure 4). SEM analysis (Figure 4A) revealed the distinct microstructural features of each component and their successful integration. The PVS membrane exhibited a smooth and uniform surface, free from cracks, pores, or phase separations, indicating the formation of a homogeneous polymer blend with strong intermolecular interactions. In contrast, the electrospun PCL10 fibers formed a continuous, bead-free, and highly aligned nanofibrous network, consistent with optimized electrospinning parameters to mimic the anisotropic architecture of Col fibrils in the native corneal stroma. The average diameter (Table 3) of the PCL nanofibers was 232 ± 44 nm, calculated from 100 random measurements, with individual diameters distributed between 150 and 300 nm (Figure 4A(b)).

Comparable findings have been reported in the literature. For instance, Türkkan et al. [66] demonstrated the fabrication of a bilayer scaffold using PCL fibers, where SEM images revealed that randomly oriented electrospun fibers formed an interconnected and highly porous network with fiber diameters ranging from 200 to 800 nm. Elkhouly et al. [67] reported a mean fiber diameter of 198 ± 32 nm for PCL fibers in bilayer scaffolds. Similarly, Zhu et al. [68] showed that coating a mineralized Col–CS film with electrospun PCL/polyvinylpyrrolidone (PVP) fibers. Their findings showed that the addition of the PCL fiber layer not only improved the interfacial adhesion between layers but also enhanced the scaffold’s surface morphology and mechanical stability. Ozdemir et al. [69] demonstrated that bilayer constructs with electrospun PCL layers had improved tensile strength and elasticity, highlighting the influence of fiber diameter and interlayer bonding on mechanical behaviour. These studies collectively confirm that PCL fibers in bilayer scaffolds improve scaffold homogeneity, surface roughness, and structural integrity, providing a biomimetic template for cellular attachment.

Furthermore, SEM micrographs (Figure 4A(c)) confirmed that the electrospun PCL fibers were deposited with a high degree of alignment, forming parallel nanofiber arrays across the membrane surface. This structural organization closely resembles the anisotropic arrangement of Col fibrils in the native corneal stroma, which is critical for guiding cell orientation and maintaining corneal transparency [70,71]. To quantitatively assess fiber orientation, ImageJ analysis was performed (Figure 4A(d)), showing a sharp distribution peak centered around 0°, indicating that most fibers were aligned along a single predominant axis. This narrow distribution confirms the reproducibility and precision of the electrospinning process in generating aligned nanofibers suitable for CSE applications [72]. Importantly, the solvent-cast PVS supporting membrane significantly enhanced fiber collection efficiency, resulting in nearly three times greater fiber distribution compared to electrospinning without the substrate. Although the PVS membrane is a dielectric polymer and thus partially insulating, its smooth surface and ability to retain surface charge locally can stabilize the electrostatic field at the collector interface. This stabilisation reduces the whipping instability of the charged jet and promotes more controlled fiber landing and deposition [73,74]. In other words, the PVS layer acts as a smooth physical scaffold with favourable electrostatic compatibility, guiding fibers toward the collection surface, minimizing loss, and supporting aligned deposition. Shao et al. [75] reported similar findings using cellulose paper substrates, highlighting the pivotal role of substrate properties in electrospinning performance. Although these substrates are electrically insulating, they can accumulate localized surface charges during fiber deposition, which partially stabilizes the electrostatic field at the collector and reduces whipping instabilities of the charged polymer jet. In addition, the smooth and uniform surface of the substrate provides physical guidance, minimizing fiber deviation and promoting alignment. Stanger et al. [76] similarly demonstrated that substrate material strongly influences fiber deposition efficiency and alignment by modulating the local electric field and providing a stable landing surface. Xue et al. [77] further explained that the combination of electrostatic stabilization and mechanical guidance enhances fiber collection density and reproducibility. Together, these effects increase scaffold homogeneity, improve biomechanical integrity, and create a more organized template for cell adhesion and proliferation, which is critical for CSE applications.

FTIR spectroscopy (Figure 4B) further confirmed the chemical composition and interactions of the bilayer scaffold components. The spectrum of the PVS membrane exhibited a broad absorption band centered around 3249 cm^−1^, attributed to O–H stretching vibrations, indicative of extensive hydrogen bonding within the semi-interpenetrating polymer network. The asymmetric stretching of carboxylate (–COO^−^) groups at 1647 cm^−1^ confirmed the ionic crosslinking of SA with calcium ions. Peaks in the 1080–1140 cm^−1^ range corresponded to C–O–C asymmetric stretching vibrations, reflecting the polymer blending of PVA and SA. Additionally, weaker bands observed at 2930 cm^−1^ were associated with C–H stretching of methylene groups in PVA [58]. The FTIR spectrum of PCL displayed sharp and well-defined peaks, characteristic of its semicrystalline polyester structure. The ester carbonyl stretching at 1724 cm^−1^ and C–O stretching vibrations at 1160–1240 cm^−1^ confirmed the presence of the ester functional groups. The CH_2_ bending and rocking vibrations at 1460 cm^−1^ and 1360 cm^−1^, respectively, further supported the semicrystalline nature of PCL. A detailed summary of all observed characteristic bands and their functional group assignments for PVA, SA, PCL, and the bilayer scaffold is provided in Table S1.

In the bilayer scaffold, the FTIR spectrum retained all major absorption bands of both PVS and PCL components without significant shifts or the appearance of new peaks, suggesting that the layers coexist through physical lamination rather than chemical bonding. This indicates that the individual functional groups of each component remain intact, preserving their chemical identities. These results are consistent with previous studies by Maheshwari et al. [78] and Huang et al. [79], who similarly reported preserving characteristic functional groups in bilayer or composite scaffolds, confirming successful structural integration without forming new chemical linkages. Overall, the FTIR analysis demonstrates that the bilayer scaffold maintains the distinct chemical features of both PCL and PVS, supporting the design strategy of combining a mechanically robust electrospun layer with a bioactive SCM.

DSC analysis (Figure 4C) was carried out to evaluate the thermal transitions of the individual layers (PVS7.5 and PCL10) and the Bilayer composite. For the solvent-cast PVS7.5 membrane, no distinct endotherm attributable to free or weakly bound water is observed near 100 °C. Instead, the thermogram displays a broad endothermic transition centered at ~225 °C, consistent with the melting of residual PVA crystalline domains and potentially overlapping with the onset of thermal degradation of the PVA/Alg blend. The breadth of this peak is consistent with reduced and heterogeneously distributed crystallinity of PVA, likely due to miscibility and molecular-level interactions with SA. In line with Miura et al. [80], PVA/Alg blends typically exhibit an upward shift in Tg with increasing Alg content, a depression of PVA’s Tm, and suppression of PVA crystallinity at higher Alg fractions. While neat PVA was not measured here, the overall thermal profile of PVS7.5 (broad, high-temperature endotherm with attenuated enthalpy) is consistent with these reported trends for miscible crystalline/amorphous polymer blends.

In contrast, the electrospun PCL10 scaffold exhibits a sharp melting endotherm near ~65 °C, characteristic of semicrystalline PCL nanofibers. The narrowness of this peak indicates the presence of well-defined crystallites, and its position aligns with prior reports for electrospun PCL [81,82,83].

The Bilayer scaffold shows two distinct endothermic transitions: a PCL-associated melting peak at ~65 °C and a broad PVA-dominated endotherm centered at ~225 °C. In addition, a minor shoulder between ~110–130 °C is visible, which may be related to partial recrystallization or relaxation phenomena within the laminated structure. No substantial shifts in the main peak temperatures are observed, confirming that lamination does not trigger chemical reactions or compromise the thermal identity of the individual layers. However, the reduced intensity of the PCL peak and the broadening of the high-temperature endotherm suggest minor interfacial interactions, local changes in crystallinity, and slight variations in effective heat capacity upon lamination. These changes do not compromise the constituent layers’ overall thermal stability or distinct thermal signatures.

Overall, the DSC results confirm that the bilayer scaffold preserves the individual thermal characteristics of PCL and PVS, while the broadening of the PVA-related peak suggests subtle polymer–polymer interactions at the interface without affecting the structural integrity of either layer. These findings indicate that each layer retains its functional properties, supporting both the mechanical robustness and the biofunctional performance of the scaffold in CSE applications.

#### 3.3.2. Surface and Environmental Interaction Analysis of the Bilayer Scaffold

**Figure 5:**
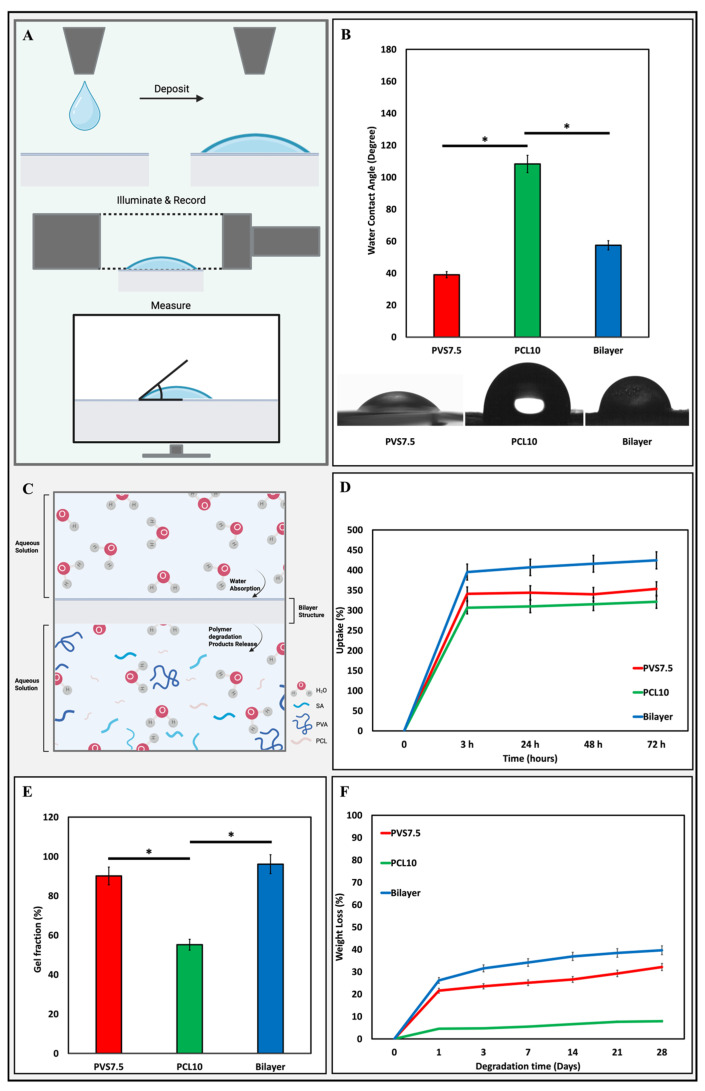
Surface Wettability, Water Affinity, and Stability Analysis of the Individual and Bilayer Scaffolds. (**A**) Representative images of microdroplet behavior on each scaffold surface, supporting the measured contact angle data. Created in BioRender. Orash, A. (2026) https://BioRender.com/a47q805 (accessed on 3 August 2026); (**B**) Static water contact angles (WCA) measurements of the PVA/SA membrane (PVS7.5), electrospun PCL10 nanofibers, and the bilayer scaffold. The PVA/SA membrane exhibits a low contact angle (39.02°), indicating high hydrophilicity, while PCL10 displays a significantly higher angle (108.31°), characteristic of hydrophobicity. The bilayer scaffold shows an intermediate angle (57.44°), reflecting improved wettability due to the presence of the underlying hydrogel layer (n = 3). (**C**) Schematic representation of scaffold interaction with aqueous environments, emphasizing the combined effects of fibrous structure and hydrogel matrix. Created in BioRender. Orash, A. (2026) https://BioRender.com/u08k413 (accessed on 3 August 2026). (**D**) Water uptake capacity of the scaffolds after immersion, showing the highest swelling in the bilayer scaffold (424.44%), followed by PVA/SA (353.25%) and PCL10 (321.19%) (n = 3). (**E**) The gel fraction values indicating crosslinking efficiency and structural stability in wet conditions, with the bilayer (96.12%) and PVA/SA (90.13%) outperforming the non-crosslinked PCL10 (55.21%) (n = 3). (**F**) In vitro biodegradation of the scaffolds after 28 days in PBS, revealing controlled degradation in the bilayer (39.65%) and PVA/SA (32.16%) compared to the more stable PCL10 (7.92%) (n = 3). * *p*  <  0.01.

The wettability and water interaction behavior of biomaterial scaffolds play a pivotal role in determining their biological performance, particularly influencing cell adhesion, proliferation, migration, and matrix secretion—key parameters for successful CSE applications [84]. In corneal environments, where transparency and hydration are tightly coupled, maintaining optimal surface energy and moisture balance is essential to mimic stromal physiology and enable stable tissue integration [85]. Static WCA measurements were thus performed to quantify the hydrophilicity of the three systems: the solvent-cast PVS7.5 membrane, the electrospun PCL10 fibrous mat, and the laminated bilayer construct (Figure 5A,B). As the solvent-cast PVS7.5 membrane in this study was fabricated under identical composition and crosslinking conditions to our previously published work, the corresponding WCA, water uptake, gel fraction, and biodegradation values were reused for consistency and comparative analysis [26]. All other physical and morphological characterizations were newly conducted for the present study.

The PVS7.5 membrane exhibited a notably low WCA (39.02°), confirming its highly hydrophilic character. This strong water affinity arises from the abundance of –OH and carboxylate (–COO^−^) groups on PVA and SA chains, which participate in intensive hydrogen bonding with surrounding water molecules [86]. Such polar functionalities not only enhance hydration but also promote protein adsorption and cell anchorage, both crucial in corneal scaffolds where optical transparency and cellular integration must coexist [87].

In contrast, the electrospun PCL10 mat displayed a significantly higher WCA (108.31°), indicative of a hydrophobic surface consistent with PCL’s aliphatic polyester backbone and limited polarity [88]. While this hydrophobicity may restrict early cell attachment and wetting, PCL remains indispensable for load-bearing applications due to its mechanical robustness, slow degradation, and electrospinnability. The bilayer scaffold, featuring a hydrophobic PCL surface laminated over the hydrophilic PVS hydrogel, exhibited an intermediate contact angle (57.44°), suggesting enhanced wettability relative to pure PCL. Although the measurement primarily reflects the outer PCL surface, moisture diffusion and capillary-driven exchange between layers likely increased effective surface energy [88]. Ahn et al. [89] reported that pure PVA exhibits a highly hydrophilic surface with a WCA of only 8.3°, reflecting the abundance of –OH groups available for hydrogen bonding with water molecules. However, the incorporation of hydrophobic PCL nanofibers into the PVA matrix significantly altered the surface characteristics. As the fraction of PCL fibers increased, the WCA progressively rose, reaching up to 64.8%. This increase can be attributed to the reduced surface availability of polar hydroxyl groups and the dominance of hydrophobic ester groups from PCL, which hinder water spreading. These findings demonstrate how the balance between hydrophilic and hydrophobic components within a composite scaffold can modulate surface wettability, a critical parameter for cell adhesion and scaffold performance [33]. This composite configuration successfully balances the hydrophilic and hydrophobic characteristics, creating a surface more favorable for cellular activities. Literature suggests that moderate wettability, typically within 40–60°, promotes optimal cell adhesion and migration, highlighting the bilayer scaffold’s enhanced suitability for CSE [90].

To further understand scaffold–fluid interactions, additional studies assessed water uptake, gel fraction, and biodegradation—critical parameters for evaluating in vivo performance and scaffold–host integration (Figure 5C–F). The bilayer scaffold demonstrated the highest water uptake capacity, reaching 424.44%, compared to 353.25% for PVS7.5 and 321.19% for PCL10 (Figure 5D), i.e., 20.2% higher than PVS7.5 and 32.1% higher than PCL10. The superior swelling of the bilayer arises from the complementary contributions of the hydrophilic hydrogel base and the interconnected, highly porous electrospun PCL mat, which increases accessible surface area and enables capillary-driven imbibition and faster diffusion pathways, thereby facilitating efficient water absorption and transport. This mechanism mirrors findings in bilayer PCL/Gel–bioactive glass scaffolds, which demonstrated significantly higher swelling rates due to increased surface area and pore volume relative to single-layer PCL scaffolds [67], and in hydrogel-hybrid electrospun fibers where swelling induced a 20–30× increase in pore volume, greatly improving fluid uptake [91]. Afzal et al. [92] reported that the relative concentrations of PVA and PCL strongly influence the swelling behavior of electrospun fibers. An increase in PVA concentration led to a higher swelling percentage, attributable to the hydrophilic nature of PVA. The greater availability of –OH groups at higher PVA content enhances water uptake through hydrogen bonding, thereby promoting greater swelling. Conversely, the incorporation of hydrophobic PCL limits water absorption, reducing the overall swelling capacity of the fibers. These findings emphasize the critical role of polymer composition in tuning the swelling properties of composite scaffolds. A similar trend is expected in bilayer system, a similarnt-cast PVA/SA layer provides a high swelling capacity owing to its hydrophilic functional groups, while the electrospun PCL layer contributes hydrophobicity and dimensional stability. Together, this layered architecture combines rapid fluid ingress (PCL) with high water retention (PVS), yielding a net swelling greater than either component alone. These swelling results should be interpreted alongside the gel fraction (Figure 5E) and biodegradation profiles (Figure 5F), collectively contextualizing network stability and resorption kinetics relevant to in vivo performance. In corneal applications, such hydration is indispensable for maintaining stromal transparency, facilitating nutrient transport, and preserving the native phenotype of resident cells.

Assessment of gel fraction, determined by solvent extraction under the conditions described in the Methods, provides a quantitative measure of the insoluble network that remains after exposure to aqueous media and therefore serves as a proxy for crosslink density and structural integrity under physiological-like conditions [93]. The bilayer construct displayed the highest gel fraction (96.12%), exceeding that of the PVS7.5 hydrogel membrane (90.13%) and markedly outperforming the PCL10 fibrous mat (55.21%) (Figure 5E). These differences reflect fundamentally different stabilization mechanisms in the three materials and directly affect handling, implantation, and in vivo persistence. For the PVS7.5 membrane, the high gel fraction is primarily attributable to effective ionic crosslinking of SA by Ca^2+^ (the well-known “egg-box” junctions), which produces a three-dimensional network resistant to aqueous extraction. Additional hydrogen-bonding and chain entanglement between PVA and Alg further reduce the extractable fraction and increase network cohesion, so that the hydrogel retains most of its mass following solvent challenge [94,95,96]. Synergistic, complementary stabilization mechanisms best explain the bilayer’s superior gel fraction relative to PVS7.5. Beyond the hydrogel’s intrinsic ionic crosslinks, the electrospun PCL layer acts as a reinforcing, interpenetrating scaffold: PCL fibers become mechanically interlocked with the hydrogel matrix during lamination and swelling, which (i) physically entraps hydrogel chains and reduces leaching of loosely bound polymer, (ii) limits large-scale network fragmentation during agitation, and (iii) provides a continuous load-bearing framework that preserves dimensional integrity [97,98]. The porous geometry of the PCL mat may also moderate swelling stresses by distributing fluid-induced strains, thereby decreasing the propensity for network rupture and mass loss. This type of fiber–hydrogel synergism, where a fibrous reinforcement increases the effective gel fraction and mechanical resilience of a hydrogel, has been reported for other hybrid scaffolds and composite dressings and underpins the practical advantage of bilayer designs for tissue engineering applications [97,98]. By contrast, the relatively low gel fraction of the PCL10 mat does not necessarily indicate chemical solubility but rather reflects the physical robustness of a non-crosslinked fibrous network under the extraction protocol used. PCL is not ionically or covalently crosslinked in our system; its integrity in aqueous media depends on fiber-fiber adhesion, packing density, and crystallinity [99,100]. Loose fiber entanglements, incomplete interfiber bonding (typical of electrospun mats), and the release of small fragments or dust during immersion/agitation can lead to an apparent reduction in retained mass despite the intrinsic water-insolubility of PCL. Moreover, slow hydrolytic degradation of PCL under prolonged conditions can contribute to mass loss over extended incubation times; however, this is a much slower process than the rapid mass changes controlled by crosslink density in hydrogels [99,100]. In summary, the high gel fraction of the bilayer arises from the combination of (i) dense ionic crosslinking within the PVS hydrogel and (ii) mechanical reinforcement and physical entrapment provided by the electrospun PCL layer. This integrated stabilization produces a construct that better resists aqueous extraction and mechanical perturbation than either component alone, a desirable feature for corneal scaffold implants where dimensional stability, controlled resorption, and retention of microarchitecture are critical.

In vitro biodegradation studies conducted over 28 days in PBS provided important insights into the scaffold’s temporal stability and resorption profile (Figure 5F). The bilayer scaffold degraded by 39.65%, which was slightly higher than the 32.16% degradation observed for PVS7.5 and significantly greater than the 7.92% reported for PCL10. This degradation pattern primarily reflects the hydrolytic sensitivity of the PVS hydrogel layer, which undergoes gradual chain scission, fragmentation, and dissolution in aqueous media [101]. In contrast, the crystalline and hydrophobic nature of PCL accounts for its slower degradation rate, as ester bond hydrolysis in PCL occurs over extended timescales, often requiring several months to years under physiological conditions [102]. The bilayer’s intermediate degradation profile is particularly advantageous for CSE, where scaffolds are required to provide temporary support while allowing for progressive ECM deposition and cellular remodeling [36]. The controlled degradation of the hydrogel component promotes deeper fluid infiltration and exposure of the scaffold’s interior, thereby enhancing nutrient diffusion and cell penetration. At the same time, the preserved PCL fiber framework ensures that mechanical integrity is maintained during the early and intermediate phases of tissue repair, preventing premature scaffold collapse. Similar findings have been reported for hydrogel–electrospun fiber composites, where the synergistic interplay between a rapidly resorbing hydrophilic phase and a more stable fibrous backbone enables both initial bioactivity and long-term dimensional stability [36,101,102]. Such a degradation profile addresses a key design requirement in corneal applications: balancing hydration and transparency with structural persistence. Too rapid degradation may result in scaffold disintegration before sufficient ECM deposition occurs, while excessively slow degradation can impede host tissue integration and remodeling [103,104,105]. The bilayer’s ability to undergo controlled and predictable biodegradation makes it a promising candidate for supporting CSE.

Collectively, these findings underscore the advantages of the bilayer design in combining the favorable physicochemical and mechanical properties of both polymer systems. Enhanced wettability, high water uptake, robust crosslinking, and a suitable degradation profile make the bilayer scaffold a promising candidate for CSE. By integrating bioactivity with mechanical integrity, the bilayer platform offers an effective strategy to mimic the native corneal microenvironment and guide functional tissue repair.

#### 3.3.3. Biomechanical, Optical, and Cytocompatibility Evaluation Relevant to Corneal Stromal Replacement

**Figure 6:**
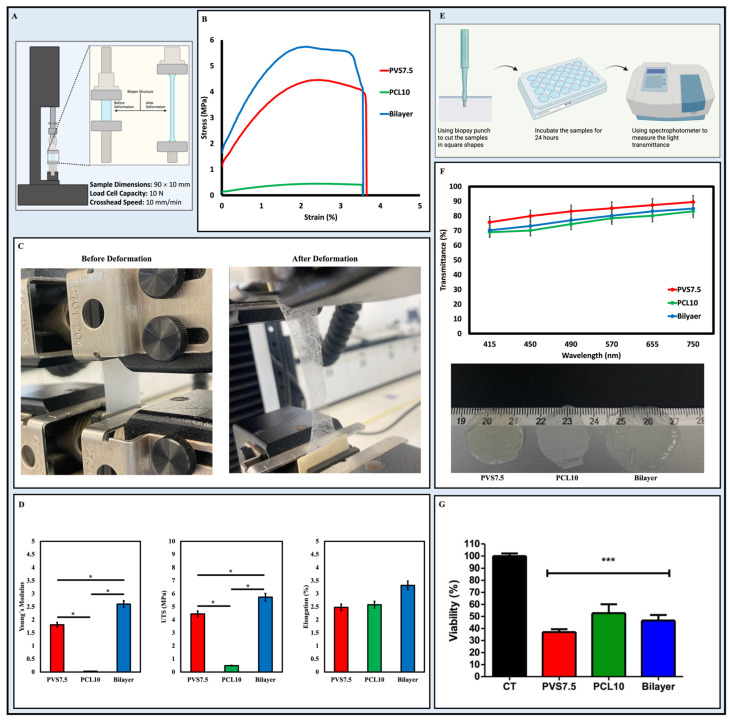
Comprehensive characterization of the bilayer scaffold. (**A**) Schematic of the mechanical testing setup used to evaluate scaffold strength. Created in BioRender. Orash, A. (2026) https://BioRender.com/e19e145 (accessed on 3 August 2026); (**B**) Representative stress–strain curves obtained from tensile tests (n = 3). (**C**) Photographs of the bilayer scaffold before and after mechanical deformation. (**D**) Quantitative analysis of mechanical properties, including Young’s modulus, UTS, and elongation at break (n = 3). * *p* < 0.01. (**E**) Schematic of the light transmittance measurement used to assess scaffold transparency. Created in BioRender. Orash, A. (2026) https://BioRender.com/i33s117 (accessed on 3 August 2026); (**F**) Light transmittance of the films measured across different wavelengths, with representative photographs showing transparency in both dry and wet conditions (n = 3). (**G**) Cell viability after 72 h exposure to the films, measured by [insert assay, e.g., MTT or live/dead assay]. Data are presented as mean ± SD, n = 5; *** *p* < 0.001 vs. CT (control cells without any film).

Maintaining appropriate mechanical properties in engineered corneal constructs is essential for ensuring long-term stability and functionality during regeneration [106]. The choice of polymers and the design of hybrid structures are therefore critical in tailoring mechanical performance to match native tissue. In the corneal stroma, mechanical integrity provides structural support, elasticity, and ocular protection, while simultaneously transmitting biomechanical cues that regulate stromal cells’ behavior and maintain stromal homeostasis [107]. Accordingly, bioengineered constructs must exhibit mechanical characteristics that approximate those of native cornea to facilitate graft integration, support keratocyte repopulation, and enable extracellular matrix remodeling during regeneration. The stress–strain profiles of PVS7.5, PCL10, and bilayer membranes are presented in Figure 6A–D, while Table 4 summarizes their corresponding Young’s modulus, UTS, and elongation at break. The Young’s modulus values were 1.81 ± 0.02 MPa for PVS7.5, 0.03 ± 0.001 MPa for PCL10, and 2.60 ± 0.20 MPa for the bilayer construct, indicating a marked enhancement in stiffness in the bilayer relative to its single-component counterparts. Similarly, UTS values were 4.45 ± 0.05 MPa (PVS7.5), 0.49 ± 0.04 MPa (PCL10), and 5.74 ± 0.02 MPa (bilayer), confirming the superior load-bearing capacity of the bilayer. The elongation at break values were 2.48 ± 0.40%, 2.58 ± 0.20%, and 3.32 ± 0.10% for PVS7.5, PCL10, and bilayer membranes, respectively, with the bilayer providing a modest but relevant improvement in extensibility.

To contextualize these findings, the mechanical properties of native human corneal stroma vary substantially due to anisotropy, hydration state, donor variability, and testing conditions, with reported Young’s modulus values ranging between 0.3 and 3 MPa [108]. Engineered scaffolds developed for CSE generally exhibit UTS values of 5–15 MPa and elongation at break between 10 and 50%, depending on polymer composition and fiber alignment [109]. Compared to these benchmarks, our bilayer construct demonstrates a Young’s modulus within the physiological range of the cornea and a UTS approaching the lower bound of reported biomimetic scaffolds, while its elongation values remain lower than those typically observed in hydrated native cornea. Nevertheless, the bilayer’s relative increase in extensibility compared to its single-layer counterparts is noteworthy, as improved flexibility may enhance resilience during surgical handling and implantation [110]. These results are consistent with prior studies showing that polymer composition and nanofiber alignment strongly influence the tensile properties of corneal substitutes [111]. Wu et al. [18], for example, demonstrated that incorporating PVA into Col-based matrices significantly improved mechanical strength, with optimized formulations achieving properties comparable to native corneal stroma. Similarly, studies on PVA/SA blends have highlighted the importance of polymer ratios, where moderate SA incorporation enhances stiffness and strength, but excessive content reduces tensile properties and elongation at break [55]. Our findings follow this trend, with the bilayer configuration balancing the high stiffness of PVA/SA with the toughness of PCL, thereby producing a synergistic reinforcement effect. Although the elongation at break of our membranes did not reach the levels reported for hydrated native cornea, the bilayer’s combination of strength, stiffness, and modest extensibility underscores its biomechanical compatibility. Importantly, too stiff constructs may impair host–graft integration and corneal transparency, whereas excessively compliant membranes may fail to provide appropriate mechanical cues for stromal cells’ phenotype maintenance and stromal remodeling [112,113]. By achieving a mechanical balance within the physiologically relevant range, the bilayer construct offers improved durability under physiological stresses such as blinking and intraocular pressure fluctuations, while maintaining structural integrity for tissue regeneration. In summary, the bilayer membrane demonstrated mechanical properties that more closely approximate native corneal stroma compared with either PVS7.5 or PCL10 alone. Its enhanced stiffness, superior tensile strength, and modestly improved extensibility highlight its suitability as a candidate for CSE. Future optimization strategies, including advanced crosslinking methods and incorporation of complementary biomaterials, may further refine its mechanical behavior while preserving optical clarity, thereby advancing its potential for clinical translation.

This section presents an evaluation and discussion of the light transmittance properties of the tested samples. Transparency is a critical parameter for engineered corneal tissues, as it directly impacts visual acuity. High light transmittance ensures that the construct permits sufficient passage of light to the retina, which is essential for maintaining clear vision. Conversely, inadequate transparency can result in visual impairment or distortion. Therefore, developing biomaterials that closely replicate the optical clarity of the native cornea is essential for cosmetic restoration and achieving functional outcomes in corneal transplantation [60]. The transmittance measurements, conducted across the 415–750 nm wavelength range, are shown in Figure 6E,F. For all samples, light transmittance generally increased with wavelength within the visible spectrum, reflecting improved optical clarity at longer wavelengths. At the wavelength of 750 nm, the PVS7.5 scaffold exhibited the highest transmittance (89.45%), followed by the bilayer construct (85.01%) and the PCL10 scaffold (83.14%). These values indicate that the PVS7.5 sample most closely approaches the transparency of the native cornea, which typically exhibits light transmittance above 90% in the visible spectrum [114].

The bilayer construct demonstrated intermediate transparency, suggesting that combining synthetic and natural polymers can improve optical properties relative to pure PCL. However, the PCL10 sample showed the lowest transmittance, which may be attributed to the intrinsic opacity of PCL due to its semicrystalline nature and light scattering at polymer chain boundaries [60]. Light transmittance in engineered corneal substitutes is governed by several interrelated factors, including polymer composition, thickness, crystallinity, surface roughness, and microstructural organization [115]. Materials with high amorphous content, such as PVA and Alg, exhibit reduced light scattering compared to semicrystalline polymers like PCL, which explains the superior optical clarity observed in PVS7.5. Additionally, the presence of hydrated polymer networks can minimize refractive index mismatches between scaffold and aqueous media, further improving transparency [116].

In contrast, the crystalline domains in PCL induce heterogeneity in the refractive index, thereby increasing scattering and decreasing overall transmittance [60]. The bilayer construct demonstrated an interesting balance: although its transmittance did not reach the level of PVS7.5, it achieved a value (85.01%) still within the range considered acceptable for corneal applications [117,118]. This result highlights the importance of material integration, as the bilayer may offer structural robustness, tunable degradation, and adequate transparency. Indeed, the combination of synthetic and natural polymers is often pursued to simultaneously optimize mechanical, biological, and optical properties [119]. It is also important to consider that achieving high transparency alone is not sufficient. Corneal substitutes must maintain optical clarity under physiological conditions, resist haze formation due to hydration changes, and preserve stability over the long term [120]. For instance, swelling behavior, ionic interactions with tear fluid, and long-term degradation may influence transmittance evolution. Thus, materials such as PVA/Alg blends, which retain water and form stable hydrogels, may provide sustained transparency in vivo [121]. When compared to the human cornea, which demonstrates more than 90% light transmittance across most of the visible spectrum [122], the PVS7.5 construct appears to be the most promising candidate, showing near-native transparency. While slightly lower, the bilayer and PCL10 scaffolds still fall within the functional threshold for engineered corneal tissue, especially if complemented by superior mechanical or biological performance. Ultimately, transparency must be considered alongside other critical design parameters, such as anisotropic Col-mimicking fiber alignment, refractive index matching, and resistance to light scattering, in order to develop clinically viable corneal implants [123]. Taken together, these findings confirm that polymer blending strategies, particularly those involving PVA and Alg, can significantly enhance the optical performance of corneal scaffolds. Meanwhile, layered or composite structures such as the bilayer construct provide an attractive compromise between transparency and functional robustness. Further optimization of scaffold architecture and polymer ratios could help achieve transparency levels indistinguishable from the native cornea while ensuring the necessary biomechanical integrity and biocompatibility for long-term success in CSE.

To evaluate the cytotoxic potential of the fabricated scaffolds, endothelial cells were cultured in contact with the films for 72 h, and cell viability was assessed using the Alamar Blue assay. As shown in Figure 6G, all fabricated scaffolds induced a reduction in viability compared to the control group (CT), which maintained close to 100% viability. The measured viability percentages were 38.94 ± 4.77% for PVS7.5, 47.35 ± 9.11% for the bilayer construct, and 52.18 ± 14.78% for PCL10. Statistical analysis confirmed that all scaffolds exhibited significantly lower cell viability compared to the control (*** *p* < 0.001).

The cytotoxicity results indicate that although the fabricated scaffolds supported measurable cellular metabolic activity, the observed viability remained substantially lower than that of the CT, indicating that the current scaffold formulations require further optimization to improve cytocompatibility. Therefore, the biological evaluation presented here should be considered a preliminary assessment of scaffold biocompatibility rather than evidence of biological readiness for CSE applications. Among the tested samples, PCL10 exhibited the highest cell viability (~52%), followed by the bilayer (~47%) and PVS7.5 (~39%). This trend suggests a clear dependence of cellular response on scaffold composition and degradation behavior. The relatively higher viability observed in PCL10 may be attributed to the lower release of ionic degradation products, as PCL is a hydrophobic polymer that degrades slowly under physiological conditions [124].

In contrast, the reduced viability in PVS7.5 may be associated with releasing ionic components (e.g., sodium and calcium from Alg) and possible osmotic stress on the cells [125]. Additionally, the higher hydrophilicity and swelling behavior of the PVA/SA layer may accelerate ion diffusion and amplify local concentration gradients, further contributing to reduced cell viability. The bilayer scaffold displayed intermediate viability, suggesting that combining PCL with PVA/SA mitigated but did not completely eliminate cytotoxic effects. This partial mitigation likely arises from the barrier effect of the PCL layer, which may slow the diffusion of ionic species while still allowing interaction with the underlying hydrophilic phase. It should also be noted that the present study was primarily designed to develop and comprehensively characterize a bilayer scaffold with appropriate structural, physicochemical, optical, and mechanical properties. Accordingly, the biological evaluation was intended as an initial screening of cytocompatibility. Although the current results demonstrate measurable cell viability, additional optimization of the scaffold composition and biological functionality will be required before considering advanced in vitro or in vivo CSE applications.

It is also important to interpret these findings within the biological context of the native corneal stroma. Unlike highly cellular tissues, the corneal stroma contains a relatively low density of quiescent keratocytes whose primary physiological function is to preserve extracellular matrix organization and corneal transparency rather than to undergo rapid proliferation. Therefore, although improving cellular viability remains an essential objective for scaffold optimization, the biological requirements of stromal substitutes differ from those of many other tissue-engineered constructs. Consequently, the present biological evaluation should be regarded as an initial assessment of scaffold biocompatibility rather than a definitive predictor of long-term stromal regeneration.

The observed viability values, ranging from approximately 39% to 52%, remain below the levels generally considered desirable for CSE, where sustained cell survival, proliferation, and extracellular matrix production are essential for successful tissue regeneration [90]. Therefore, while the present scaffold demonstrated favorable transparency, wettability, mechanical integrity, and structural organization, these physicochemical advantages alone are insufficient to establish biological suitability. Accordingly, the current findings should be interpreted as demonstrating the feasibility of the bilayer scaffold design rather than confirming its biological optimization. Moreover, it should be emphasized that the present study focused primarily on establishing the structural and physicochemical feasibility of the bilayer scaffold. Consequently, additional biological optimization—including improved cell adhesion, viability, and long-term cellular functionality—represents the next stage in the development of this platform.

Several factors such as residual solvent traces, ionic crosslinking density, and degradation byproducts may contribute to reduced cell viability. Previous studies have shown that tuning calcium concentrations in Alg-based scaffolds can regulate cell survival and proliferation by minimizing ionic stress while maintaining structural stability [126]. Accordingly, fine-tuning the crosslinking strategy in the PVA/SA layer represents a promising route to improve cytocompatibility in future designs. In addition, the absence of biological signaling molecules or extracellular matrix-derived adhesion motifs within the current scaffold formulation may also have contributed to the limited cellular response. Electrospun PCL provides excellent mechanical support but possesses an intrinsically hydrophobic surface, while the PVA/SA hydrogel lacks specific cell-recognition sequences capable of actively promoting cell adhesion and proliferation. Consequently, although the bilayer architecture successfully reproduces several structural characteristics of the native corneal stroma, further biological functionalization is expected to be necessary to improve cell-scaffold interactions. Furthermore, the electrospun PCL layer was primarily incorporated to provide structural reinforcement and reproduce the anisotropic architecture of the native stromal lamellae rather than to function as a biologically active surface. Likewise, the solvent-cast PVA/SA layer was designed to provide hydration, transparency, and mechanical support. Future incorporation of extracellular matrix-derived molecules, bioactive natural polymers, or peptide-based adhesion motifs is therefore expected to transform this structurally optimized scaffold into a biologically more favorable microenvironment capable of supporting enhanced cellular attachment and long-term tissue regeneration. Interestingly, in our previous study, the incorporation of Aloe vera extract into electrospun PCL scaffolds increased cell viability to values exceeding 90%, demonstrating that appropriate biological functionalization can substantially enhance cellular responses without compromising scaffold integrity [29]. These findings suggest that the relatively low viability observed in the present work is unlikely to represent an inherent limitation of the bilayer design itself but rather reflects the need for further optimization of its biological composition.

Building on this evidence, future work on the present scaffolds could involve the incorporation of AV or similar natural polymers to counterbalance cytotoxic effects while preserving optical and mechanical performance. Overall, the present biological results demonstrate that the developed bilayer scaffold possesses a robust structural and physicochemical foundation but requires further biological optimization before it can be considered a fully developed CSE scaffold. Future investigations will therefore focus on optimizing polymer composition, crosslinking density, degradation behavior, and incorporating bioactive molecules such as Aloe vera, extracellular matrix-derived proteins, or peptide-based adhesion motifs to improve cytocompatibility while preserving the favorable optical and mechanical properties demonstrated in this study.

## 4. Conclusions

In this work, we successfully developed and systematically characterized a bilayer scaffold composed of electrospun PCL nanofibers over a solvent-cast PVS membrane for CSE. Both individual layers and the bilayer construct were thoroughly evaluated, covering morphological, physicochemical, thermal, mechanical, optical, and biological properties. The PVS membrane demonstrated a smooth and crack-free structure with stable thermal transitions, while the PCL fibers exhibited a uniform, bead-free, and highly aligned nanofibrous morphology. When integrated, the bilayer scaffold preserved both layers’ structural integrity and functional signatures, highlighting good interfacial stability. Functionally, the construct achieved desirable wettability, high water uptake and gel fraction, controlled biodegradation, and mechanical performance aligned with the requirements of corneal tissue.

Furthermore, it maintained high transparency, whereas the preliminary biological evaluation identified the need for further optimization to enhance cellular viability despite the favorable structural and physicochemical characteristics of the scaffold. Considering that the native corneal stroma is a relatively hypocellular tissue, the present biological assessment should be interpreted as an initial evaluation of scaffold biocompatibility rather than a demonstration of complete biological functionality. The comprehensive characterization from the casting membrane to the electrospun fibers and finally to the bilayer construct demonstrates the robustness and versatility of this design. Compared with previously reported PVA-based and composite corneal scaffolds, the present study does not propose an entirely new fabrication technique but rather advances the field through the integration of a solvent-cast PVA/SA membrane with highly aligned electrospun PCL nanofibers into a biomimetic bilayer architecture. Furthermore, the systematic evaluation of the individual layers and the integrated construct across morphological, physicochemical, thermal, optical, mechanical, degradation, and preliminary biological aspects provides a comprehensive framework for understanding the structure–property relationships governing scaffold performance in CSE. These findings support the bilayer scaffold as a promising structural and physicochemical platform that balances mechanical stability, transparency, and interfacial integrity while providing a foundation for further biological optimization. The present work therefore establishes a robust structural and physicochemical platform for CSE, while highlighting that biological optimization through improved cytocompatibility and biofunctionalization constitutes the next critical step toward clinical translation. Although the present scaffold demonstrated encouraging structural, optical, and mechanical properties, the observed cellular viability indicates that further refinement of the scaffold formulation is necessary before considering advanced preclinical or clinical applications. Future studies will focus on enhancing the biological performance of this system by optimizing the polymer composition and crosslinking strategy while incorporating bioactive molecules, extracellular matrix-derived components, natural polymers, or surface functionalization strategies to further improve cellular viability, proliferation, and differentiation without compromising the scaffold’s favorable optical and mechanical properties. Integrating advanced fabrication techniques such as coaxial electrospinning or 3D bioprinting may provide greater control over microarchitecture and cellular interactions. Together, these advancements are expected to facilitate the continued development of the PCL–PVS bilayer scaffold toward a biologically optimized platform for future CSE applications and, ultimately, clinical translation.

## Figures and Tables

**Table 1:** Mechanical behavior of the casting films at different polymer concentrations (* *p* < 0.01, n = 3).

Samples	Thickness (μm)	Young’s Modulus (MPa)	UTS (MPa)	Elongation (%)
PVS5	92 ± 6	0.97 ± 0.01	2.42 ± 0.01	2.31 ± 0.01
PVS7.5	106 ± 7	1.81 ± 0.02	4.45 ± 0.05 *	2.48 ± 0.4
PVS10	121 ± 7	1.52 ± 0.01	3.82 ± 0.01	2.50 ± 0.01

**Table 2:** Average fiber diameters and mechanical properties of electrospun PCL scaffolds at different polymer concentrations (* *p* < 0.01, n = 3).

Samples	Mean Fiber Diameter (nm)	Young’s Modulus (MPa)	UTS (MPa)	Elongation (%)
PCL7.5	185 ± 43	0.005 ± 0.001	0.02 ± 0.001	2.97 ± 0.1
PCL10	215 ± 48	0.03 ± 0.001 *	0.49 ± 0.01 *	2.58 ± 0.2
PCL12.5	291 ± 42	0.008 ± 0.001	0.29 ± 0.01 *	2.11 ± 0.1

**Table 3:** Average fiber diameters of PVS7.5, PCL10, and Bilayer scaffolds (n = 3).

Samples	Mean Fiber Diameter (nm)
PVS7.5	-
PCL10	215 ± 48
Bilayer	232 ± 44

**Table 4:** Mechanical properties of PVS7.5, PCL10, and Bilayer scaffolds: Young’s modulus, UTS, and elongation at break (* *p* < 0.01; n = 3).

Samples	Young’s Modulus (MPa)	UTS (MPa)	Elongation (%)
PVS7.5	1.81 ± 0.02 *	4.45 ± 0.05 *	2.48 ± 0.4
PCL10	0.03 ± 0.001	0.49 ± 0.01	2.58 ± 0.2
Bilayer	2.60 ± 0.20 *	5.74 ± 0.02 *	3.32 ± 0.1
Cornea	~0.3–3	~5–15	~10–50

## Data Availability

The original contributions presented in this study are included in the article/Supplementary Material. Further inquiries can be directed to the corresponding authors.

## Supplementary Materials

The following supporting information can be downloaded at https://www.mdpi.com/article/10.3390/polym18151928/s1: Table S1. Characteristic FTIR absorption bands and corresponding functional groups of PVA, SA, PCL, and the bilayer scaffold.
