# Supplementary material for: Bilayer Scaffold for Corneal Stromal Engineering: Solvent-Cast Polyvinyl Alcohol/Sodium Alginate and Electrospun Aligned Polycaprolactone Fibers
Source: Polymers (Basel). 2026 Aug 6;18(15):1928. doi: 10.3390/polym18151928 (PMC13468754; doi:10.3390/polym18151928)
Supplement: Supplementary file 1 [file polymers-18-01928-s001.zip › polymers-4433911-supplementary.pdf]

Table S1. Characteristic FTIR absorption bands and corresponding functional groups of PVA, SA, PCL, and the bilayer scaffold.

**Wavenumber (cm^{-1})**	**Functional group / Vibration mode**	**Assignment / Polymer component**
3240–3300	O–H stretching (hydrogen-bonded hydroxyl groups)	PVA, SA
2930	C–H stretching (–CH_2– asymmetric)	PVA, PCL
1720–1730	C=O stretching (ester carbonyl)	PCL
1640–1650	Asymmetric stretching of –COO^- (carboxylate group)	SA (Ca^{2+} crosslinking)
1460	CH_2 bending (scissoring)	PCL
1360	CH_2 rocking vibration	PCL
1250–1160	C–O–C asymmetric stretching (ester linkage)	PCL
1140–1080	C–O–C and C–O stretching (acetate and ether linkages)	PVA/SA blend
840–880	C–C stretching (crystalline PCL phase)	PCL
620–640	Na–O or Ca–O vibration (ionic crosslinking)	SA (crosslinked regions)
